# Microalbuminuria (Moderate Albumin Excretion) and its relationship with Silent myocardial ischemia in treatment naïve type II diabetic patients

**DOI:** 10.12669/pjms.36.3.938

**Published:** 2020

**Authors:** Qurban Hussain, Mulazim Hussain Bukhari, Faiza Afzaal, Wajiha Fatima

**Affiliations:** 1 Dr. Qurban Hussain, MBBS, FCPS Medicine. Senior Registrar Medicine, Jinnah Hospital, Lahore, Pakistan; 2 Prof. Mulazim Hussain Bukhari, MBBS, DCP, MPhil, FCPS, PhD. Professor of Pathology and Head of Pathology UCMD, University of Lahore, Pakistan; 3 Dr. Faiza Afzaal, MBBS, MCPS Anesthesia. Post Graduate Trainee FCPS Anesthesia, Jinnah Hospital, Lahore, Pakistan; 4 Dr. Wajiha Fatima, MBBS, FCPS Medicine. Senior Registrar Medicine, Jinnah Hospital, Lahore, Pakistan

**Keywords:** Diabetes mellitus, Microalbuminemia, Moderate Albumin excretion, Myocardial Infarction, Silent myocardial ischemia

## Abstract

**Objective:**

To determine the frequency of microalbuminuria (MAU) or Moderate Albumin Excretion (MAE) in treatment naïve type II diabetic patients and to compare the frequency of silent myocardial ischemia in treatment naive Type-II diabetic patients with and without microalbuminuria.

**Methods:**

It was a cross sectional survey conducted in the outpatient Department, Jinnah Hospital Lahore, from 30th May 2015 to 29th November 2015. There were 227 patients, (consecutive treatment naïve type II diabetic patients), presenting to outpatient department were enrolled in the study. MAU/MAE, silent myocardial ischemia and effect modifiers like HbA1C > 7%, smoking pack years and dyslipidemia was determined. MAU/MAE was determined by urinary albumin excretion rate of 30-300 mg/24 hours and included patients underwent exercise tolerance test to diagnose silent myocardial ischemia.

**Results:**

Out of total 165 patients (72.7%) were male and remaining 62 patients (27.3%) were female. The 54 patients (23.8%) had MAU/MAE. The 44 patients (19.4%) had silent myocardial infarction. When we cross tabulated microalbuminuria with silent myocardial infarction, result were significant. Out of 54 patients with MAU/MAE, 16 cases had silent myocardial infarction.

**Conclusion:**

The frequency of microalbuminuria/ Moderate Albumin Excretion in treatment naïve type II diabetic patients was high and associated with the frequency of silent myocardial ischemia in treatment naïve type II diabetic patients with and without MAU MAU/MAE.

## INTRODUCTION

Myocardial infarction (MI) is a leading cause of early mortality throughout the world causing 9.4 million deaths each year globally.^1^ Type-II diabetes is a growing health problem associated with high rates of cardiovascular morbidity and mortality.^2^-^4^

Individuals with diabetes have a higher prevalence of silent myocardial ischemia than those without diabetes, and diabetic patients without prior myocardial infarction have as high a risk of death from coronary disease as nondiabetic patients with previous myocardial infarction. The criteria of SMI was presence of objective evidence of myocardial ischemia in the absence of chest discomfort or another anginal equivalent symptom (e.g., dyspnea, nausea, diaphoresis, etc). Objective evidence of silent myocardial ischemia may be obtained by ST segment changes on EEK, consistent with ischemia seen during exercise treadmill testing or ambulatory monitoring.^5^-^7^

In comparison to nondiabetic patients, diabetic patients have a lower incidence of one-vessel disease and a higher incidence of three-vessel/left main artery disease and left ventricular dysfunction. Most importantly, myocardial ischemia is often asymptomatic in patients with diabetes until the onset of myocardial infarction or cardiac death.^8^

Rationale of current study was that our population differs from other in health seeking behavior and attitude so delay in diabetes diagnosis is common leading to early complications. Current study therefore determined the role of MAU in treatment naive patients and help to subsequently reduce morbidity and mortality associated with it because no study is available in our set up showing the significance of MAU in treatment naive diabetic patients regarding development of silent MI on online search engines. The available study in local research repository of role of MAU in early detection of silent MI has not considered diabetes control and it is eight-year old.^10^ MAU is a possible risk factor for SMI in Type-II DM. In a study it has been seen that urinary MAU can be used particularly as a screening test for early detection of SMI.^9^ Current study will therefore determine the role of micro albuminuria in treatment naive patients and help to subsequently reduce morbidity and mortality associated with it.

## METHODS

After the approval ethical committee (Ref. No: CPSP/REU/MED-2012-055-7496, dated on May 29, 2015), a cross sectional study was carried out in Outpatient department, Jinnah Hospital Lahore from 30th May 2015 to 29th November 2015. A sample size of 227 was calculated with 95% confidence interval and 80% power of study, taking proportion of MAU in DM about 18%.^8^ Through a Non-probability / consecutive sampling subjects between 30–60 years of age of either gender and treatment naïve type II diabetic defined as Fasting plasma glucose concentration >126mg/dl (7.0mmole/L) in patients with age > 30 years with negative history of previous diagnosis and use of oral hypoglycemic with urinary albumin excretion rate of 30-300mg/24 hours were included. Normoalbuminuria was defined as a urinary albumin excretion < 30 mg/d and microalbuminuria was defined as a urinary albumin excretion between 30 and 300 mg/d.^9^

Silent myocardial ischemia (MI) was determined with the help of treadmill exercise tolerance test (ETT). According to Bruce Protocol, an ischemic ECG response to exercise test was defined by presence of both at least 1 mm horizontal ST segment depression and 1.5mm down sloping ST segment depression measured at the J point. Subjects with history of angina pectoris, previous coronary angiography or coronary artery revascularization, MI or heart failure and evidence of Q-wave myocardial infarction, ischemic ST-segment or T-wave changes were excluded. Data was collected on a structured questionnaire (proforma) containing background information like age, sex and exposure/outcome measures and effect modifiers like HbA1C > 7%, smoking pack years and dyslipidemia.

MAU/MAE was determined by urinary albumin excretion rate of 30-300 mg/24 hours collection by standard laboratory technique by Hitachi analyzer 912. Included patients underwent exercise tolerance test to diagnose silent myocardial ischemia. SMI was compared in patients with and without micro albuminuria. Data collected was entered and analyzed in the SPSS version 17.

Mean with standard deviation was calculated for quantitative variables like age and frequency and percentages in case of categorical variables like gender, micro albuminuria and silent myocardial ischemia. Data was stratified for HbA1C > 7%, smoking pack years > 10 and presence of dyslipidemia. Both groups and post stratification difference was compared using chi square test to determine the significant difference. The p value <0.05 was taken as significant

## RESULTS

In our study population 227 patients were included with mean age of 52 ± 6.607 ranged from 40 to 60 years. The 100 patients (44.1%) in our study population were below 50 years whereas 127 patients (55.9%) were either 50 years or more in age. In the stydy,165 patients (72.7%) were male and remaining 62 patients (27.3%) were female. The 54 patients (23.8%) had MAE. The 111 patients (48.9%) had HbA1C above 7% and 41 patients (18.1%) had family history of DM. Only nine patients (8.4%) had dyslipidemia while remaining 208 patients was not having dyslipidemia. (Table-I)

**Table-I:** Descriptive analysis of age, gender, family history, presence of Microalbuminemia and dyslipidemia association of Diabetes, mellitus.

Variables (n=227)	Frequency	Percentage
*Age Mean=52.00 SD±6.62*		
< 50 years	100	44.1
> 50 years	127	55.9
*Gender*		
Male	156	72.7
Female	62	26.3
*Microalbuminemia/Moderate Albumin Excretion*		
Yes	54	23.8
No	173	76.2
*HbA1C > 7%*		
Yes	111	48.9
No	116	51.1
*Family history of DM*		
Yes	41	18.1
No	186	81.9
*Dyslipidemia*		
Yes	19	8.4
No	208	91.6
*Family history of MI*		
Yes	129	56.8
No	98	43.2
*Silent Myocardial infarction*		
Yes	44	19.4
No	183	80.6
*BMI >30kg/m2*		
Yes	124	54.6
No	103	45.4
*DM > 1*		
Yes	19	8.4
No	208	91.6

The 23 patients of our sampled population had history of smoking more than 10 pack a year. There were 129 patients (56.8%) with family history of myocardial infarction and 44 patients (19.4%) had silent myocardial infarction. In the study,124 patients (54.6%) had body to mass index (BMI) more than 30 Kg/m^2^ and 19 patients (8.4%) had duration of DM greater than one year. (Table-I-II)

**Table-II:** Micro albuminuria and silent myocardial infarction.

	Microalbuminuria	Silent Myocardial infarction		P value

		Yes	No	
Micro albuminuria	Yes	16 (29.6%)	38 (70.4%)	0.033
	No	28 (16.2%)	145 (83.8%)	

When we cross-tabulated MAU with silent myocardial infarction, and applied Pearson chi square test, result came up significant (p=0.03). Out of 54 microalbuminuria patients 16 were having silent myocardial infarction. When we stratified data of age regarding Silent Myocardial infarction among patients with and without MAE, the results came up non-significant for both age groups. When we stratified data of gender regarding Silent Myocardial infarction among patients with and without MAE, the results came up significant for male (p=0.007) and non-significant for female (p=0.735). (Table-III)

**Table-III:** Micro albuminuria and silent myocardial infarction and risk factors cross tabulation.

	Microalbuminuria/ Moderate Albumin Excretion	Silent Myocardial infarction		P-value

		Yes	No	
Age < 50	Yes	4	10	0.214
	No	13	73	
Age > 50	Yes	12	28	0.103
	No	15	72	
Male	Yes	14	25	0.007
	No	20	106	
Female	Yes	2	13	0.735
	No	8	39	
HbA1C > 7 %	Yes	6	20	0.536
	No	15	70	
HbA1C < 7%	Yes	10	18	0.015
	No	13	75	
Family History	Yes	3	10	0.695
	No	5	23	
Dyslipidemia	Yes	1	6	0.891
	No	2	10	
Smoking	Yes	2	9	0.924
	No	2	10	
Family h/o MI	Yes	10	16	0.020
	No	18	85	
BMI > 30	Yes	7	16	0.016
	No	11	90	
DM > 1 year	Yes	1	5	0.554
	No	1	12	

When we stratified HbA1C >7% regarding Silent Myocardial infarction among patients with and without MAE, the results came up significant for those having HbA1C below 7% with p value 0.015 and non-significant for HbA1C >7% (p=0.735). Family history of DM regarding Silent Myocardial infarction among patients with and without MAE was cross tabulated and the results came up significant for those having no family history of DM (p=0.023) and among patients with and without MAE, the results came up significant for those having negative results for dyslipidemia (p=0.017) while non-significant among dyslipidemia patients (p=0.891). Family history of MI regarding SMI among patients with and without MAU, also showed a significant relationship (p=0.02). BMI >30Kg/m^2^ and DM of more than one year duration showed a significant relationship. (P < 0.05).

## DISCUSSION

Early detection of Microalbuminuria/Moderate albumin excretion in asymptomatic patients, can be beneficial in leading to complicated cardiovascular diseases and may be helpful by early diagnosis of this high risk disease.^8^,^9^

There are multiple biomarkers for the diagnosis of CVD as a potential screening tools but MAU is one that has shown promising results. This is a marker, considered to be associated with oxidative stress in various ischemic and non-ischemic processes of the cardiovascular diseases in patients of diabetes mellitus.^10^-^12^ Many studies have shown that presence of MAU is a significant predictor of myocardial ischemia.^10^-^12^

The presence of MAU in Type-2 DM is considered a potential risk for developing progressive renal diseases as well as proving as a diagnostic marker of predilection for generalized cardiovascular disease.^13^,^14^ In our study, 23.8% of treatment naive Type-II diabetic patients had MAU. The 44 patients (19.4%) had silent myocardial infarction. When we cross-tabulated MAU with silent myocardial infarction, and applied Pearson chi square test, result came up significant (p=0.03). Out of 54 MAU patients 16 were having silent MI. This frequency is quite high but comparable with other studies in other countries. In one study, 25 of the 77 asymptomatic DM patients (32 %) had ischemia. MAU was present in 18% patients.^8^ Ten percent without ischemia had MAU and 32% with ischemia had MAU (i.e. 16.9%, 13/77). Only MAU was found as a significant predictor of MI (OR 4.42, 95% CI 1.27 – 15.40; P = 0.019). No statistically significant trend (P < 0.1) was observed for any other variable.^8^ An Iraqi study, published eight years back had shown silent myocardial ischemia 30% & 6% in diabetic patient with and without micro albuminuria.^10^-^13^. Our findings are not consistent with Bilgi et al. who could not find any association of microalbuminuria of diabetic nephropathy and CVD.^14^ In another study, by Yurtdas et al., it was found that there was a significant inverse association between MAU, and Silent MI in treatment naive Type-II DM patients and even in non diabetic patients.^15^,^16^

### Limitation of the study

The current study is its small sample size and population selection from a tertiary care hospital, which is not representative of our total population.

## CONCLUSION

The frequency of MAU/MAE in treatment naive Type-II diabetic patients was high and the frequency of silent myocardial ischemia in treatment naive Type-II diabetic patients was also high in patients of DM without MAU/MAE.

### Recommendations

The treatment naive Type-II diabetic patients should be screened for urine albumin excretion test (spot urine albumin creatinine ratio for convenience). MUA should be used early marker of detection of silent myocardial ischemia.

### Author`s Contribution:

**QH** did data collection, statistical analysis and manuscript writing and conceived, designed, and edited the manuscript.

**MHB** Conceived, designed, did review, final approval of manuscript and is responsible for integrity of research.

**FA, WF** helped in collection of data writing the manuscript.

## References

[1] World Health Organization. Cardiovascular diseases (CVDs) Fact sheet N°317.

[2] Bourque JM, Patel CA, Ali MM, Perez M, Watson DD, Beller GA (2013). Prevalence and predictors of ischemia and outcomes in outpatients with diabetes mellitus referred for single-photon emission computed tomography myocardial perfusion imaging. Circ Cardiovas Imaging.

[3] Gui MH, Li X, Lu ZQ, Gao X (2013). Fasting plasma glucose correlates with angiographic coronary artery disease prevalence and severity in Chinese patients without known diabetes. Acta Diabetol.

[4] Hage FG, Lusa L, Dondi M, Giubbini R, Iskandrian AE (2013). Exercise stress tests for detecting myocardial ischemia in asymptomatic patients with diabetes mellitus. Am J Cardiol.

[5] Marciano C, Galderisi M, Gargiulo P, Acampa W, D'Amore C, Esposito R (2012). Effects of type 2 diabetes mellitus on coronary microvascular function and myocardial perfusion in patients without obstructive coronary artery disease. Eur J Nucl Med Mol Imaging.

[6] Presotto L (2013). Effects of type 2 diabetes mellitus on coronary microvascular function and myocardial perfusion in patients without obstructive coronary artery disease. Eur J Nucl Med Mol Imaging.

[7] Raza SM (1987). Silent Myocardial Ischemia. J Pak Med Assoc.

[8] Giovacchini G, Cappagli M, Carro S, Borrini S, Montepagani A, Leoncini R (2013). Microalbuminuria predicts silent myocardial ischaemia in type 2 diabetes patients. Eur J Nucl Med Mol Imaging.

[9] Hussain AR, Khan MA, Ali AK, Vaales S (2005). Type 2 diabetes in rural and urban population:diverse prevalence and associated risk factors in Bangladesh. Diabetes Med.

[10] Nadir MA, Rekhraj S, Wei L, Lim TK, Davidson J, MacDonald TM (2012). Improving the primary prevention of cardiovascular events by using biomarkers to identify individuals with silent heart disease. J Am Coll Cardiol.

[11] Hussein AZF, Strak SK (2006). Silent myocardial ischemia and microalbuminuria in asymptomatic Type-2 Diabetic patients. Pak J Med Sci.

[12] Swoboda PP, McDiarmid AK, Erhayiem B, Ripley DP, Dobson LE, Garg P (2017). Diabetes Mellitus, Microalbuminuria, and Subclinical Cardiac Disease:Identification and Monitoring of Individuals at Risk of Heart Failure. J Am Heart Assoc.

[13] de Boer IH, Rue TC, Cleary PA (2011). Long-term renal outcomes of patients with type 1 diabetes mellitus and microalbuminuria:an analysis of the Diabetes Control and Complications Trial/Epidemiology of Diabetes Interventions and Complications cohort. Arch Intern Med.

[14] Bilgi M, Keser A, Katlandur H, Sahin E, Kalkan AO, Yildiz M (2017). Evaluation of the Relationship Between Microalbuminuria and Urine Ischemia-Modified Albumin Levels in Patients with Diabetic Nephropathy. J Clin Lab Anal.

[15] Zabeen S, Hoque MM, Rahman MR (2012). Silent Myocardial Ischemia (SMI) and its Association with Micr oalbuminuriain Type 2 Diabetes Mellitus (DM). BSMMU J.

[16] Yurtdas M, Ozdemir M, Aladag N, Yaylali YT (2017). Association of Heart Rate Recovery with Microalbuminuria in Non-Obstructive Coronary Artery Disease. Cardiol Res.

